# Poverty-Related Diseases Attack Simultaneously

**DOI:** 10.4269/ajtmh.15-0823

**Published:** 2016-05-04

**Authors:** Yves Thierry Barogui, Ymkje Stienstra

**Affiliations:** Centre de Dépistage et de Traitement de l'Ulcère de Buruli de Lalo, Bénin; Department of Internal Medicine/Infectious Diseases, University Medical Center Groningen, University of Groningen, Groningen, The Netherlands

**Figure 1.**
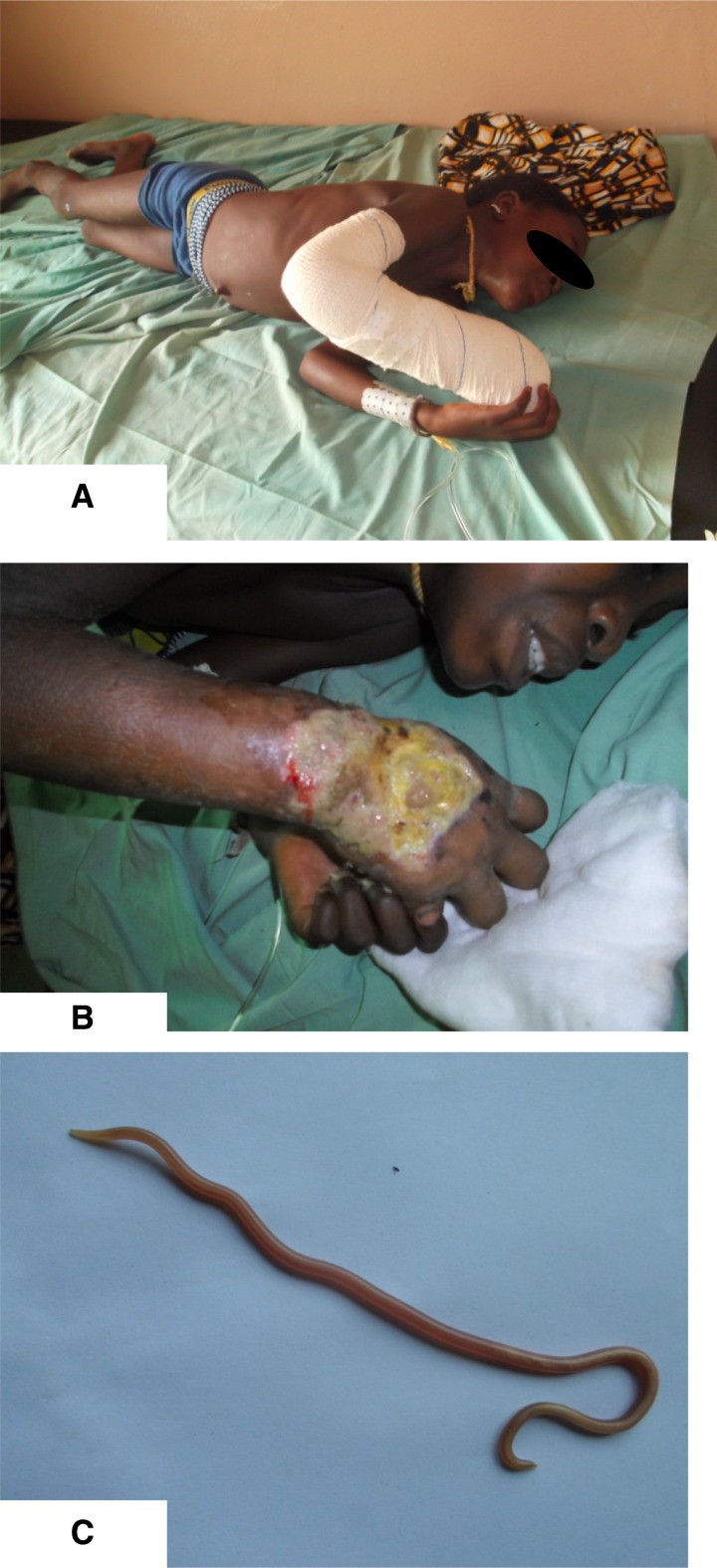
Poverty-related diseases attack simultaneously; tetanus, buruli ulcer and ascariasis.

A 10-year-old girl was admitted in Benin (West Africa) with trismus, irritability, and fever. Physical examination revealed clinical findings compatible with tetanus, including a stiff neck, opisthotonus, risus sardonicus, and dysphagia (Figure 1A

). On her right arm, she had an ulcer for 2 months. By IS2404 polymerase chain reaction,^1^ the ulcer was confirmed as Buruli ulcer, caused by *Mycobacterium ulcerans*, a neglected tropical disease (Figure 1B). The ulcer was treated with a black powder by a local traditional healer. We believe this procedure may have introduced the *Clostridium tetani* spores because 1 year ago, another Buruli ulcer patient presented with tetanus after a similar traditional treatment. The patient was treated in a quiet, dark room with equine antitoxin, metronidazol, and diazepam. She did not receive tetanus vaccinations in the past. The Buruli ulcer was initially treated with wound care and 8 weeks of rifampicin and streptomycin. Nine days after admission, an *Ascaris lumbricoides* crawled out of the patient's nose (Figure 1C). Adult worms tend to migrate in the presence of anesthetics or fever.^2^ We suspect her generalized tetanic spasms to have directed this worm toward her nose. Twenty-two days after admission, the generalized tetanic spasms abated. The ascariasis was treated with albendazole.

The patient was discharged 10 months after admission because of a complicated treatment of the Buruli ulcer consisting of multiple debridements and skin grafting. There were no signs of osteomyelitis. This patient unfortunately has permanent functional limitations of her wrist as a consequence of Buruli ulcer. Permanent functional limitations are common in former Buruli ulcer patients, especially in patients with large ulcers.^3^

These three panels illustrate the fact that poverty-related diseases are coendemic and may lead to a synergistic threat to a persons' health.
